# Pharmacokinetic/Pharmacodynamic Analysis of Tedizolid Phosphate Compared to Linezolid for the Treatment of Infections Caused by Gram-Positive Bacteria

**DOI:** 10.3390/antibiotics10070755

**Published:** 2021-06-22

**Authors:** Alicia Rodríguez-Gascón, Amaia Aguirre-Quiñonero, María Angeles Solinís Aspiazu, Andrés Canut-Blasco

**Affiliations:** 1 Pharmacokinetic, Nanotechnology and Gene Therapy Group (PharmaNanoGene), Faculty of Pharmacy, Lascaray Research Centre, University of the Basque Country UPV/EHU, 01006 Vitoria-Gasteiz, Spain; alicia.rodriguez@ehu.eus (A.R.-G.); marian.solinis@ehu.eus (M.A.S.A.); 2 Instituto de Investigación Sanitaria Bioaraba, 01009 Vitoria-Gasteiz, Spain; amaia.aguirrequinonero@osakidetza.eus; 3 Microbiology Service, Araba University Hospital, Osakidetza Basque Health Service, 01009 Vitoria-Gasteiz, Spain

**Keywords:** tedizolid, linezolid, PK/PD, acute bacterial skin and skin-structure infections

## Abstract

Tedizolid and linezolid have antibacterial activity against the most important acute bacterial skin and skin-structure infection (ABSSSIs) pathogens. The objective of this work was to apply PK/PD analysis to evaluate the probability of attaining the pharmacodynamic target of these antimicrobials based on the susceptibility patterns of different clinical isolates causing ABSSSI. Pharmacokinetic and microbiological data were obtained from the literature. PK/PD breakpoints, the probability of target attainment (PTA) and the cumulative fraction of response (CFR) were calculated by Monte Carlo simulation. PTA and CFR are indicative of treatment success. PK/PD breakpoints of tedizolid and linezolid were 0.5 and 1 mg/L, respectively. Probability of treatment success of tedizolid was very high (>90%) for most staphylococci strains, including MRSA and coagulase-negative staphylococci (CoNS). Only for methicillin- and linezolid-resistant *S. aureus* (MLRSA) and linezolid resistant (LR) CoNS strains was the CFR of tedizolid very low. Except for LR, daptomycin-non-susceptible (DNS), and vancomycin-resistant (VRE) *E. faecium* isolates, tedizolid also provided a high probability of treatment success for enterococci. The probability of treatment success of both antimicrobials for streptococci was always higher than 90%. In conclusion, for empiric treatment, PK/PD analysis has shown that tedizolid would be adequate for most staphylococci, enterococci, and streptococci, even those LR whose linezolid resistance is mediated by the *cfr* gene.

## 1. Introduction

Linezolid, the first commercialized oxazolidinone antibiotic, has activity against a wide variety of Gram-positive bacteria, including methicillin-resistant *Staphylococcus aureus* [1]. Regardless of the patient’s hepatic or renal function, the authorized dose of linezolid is 600 mg every 12 h. Tedizolid phosphate is the second commercially available oxazolidinone antibiotic, with antibacterial activity against the most important acute bacterial skin and skin-structure infection (ABSSSIs) pathogens, including some linezolid-resistant *Staphylococcus aureus* and enterococci [2]. Tedizolid, like linezolid, inhibits bacterial protein synthesis by binding to 23S rRNA of the 50S subunit of the ribosome. It has been approved in several countries, including the United States, the European Union, and Canada [3].

Although it is similar to linezolid, tedizolid has a modified side chain at the C-5 position of the oxazolidinone nucleus that confers activity against some linezolid-resistant strains and possesses an optimized C- and D-ring system that increases potency through additional binding site interactions. Tedizolid offers theoretical advantages: on the one hand, it is dosed once daily [4]; on the other hand, it is a more potent protein synthesis inhibitor, enabling it to overcome chloramphenicol-florfenicol resistance mechanisms (*cfr* gene) [5]. Like linezolid, the dose of tedizolid does not need to be modified in patients with renal impairment, hepatic impairment, on hemodialysis, or whenever switching from intravenous to oral administration [6].

Tedizolid is four- to eightfold more potent in vivo than linezolid against all species of staphylococci, enterococci, and streptococci, including drug-resistant phenotypes such as methicillin-resistant *S. aureus* (MRSA) and vancomycin-resistant enterococci (VRE) and linezolid-resistant (LR) phenotypes whose linezolid resistance is mediated by the *cfr* gene [7]. The *cfr* gene, due to its ubiquity, plays an important role in the spread of drug resistance. In terms of drug resistance mechanism, the *cfr* gene confers resistance through a non-mutated mechanism, while linezolid resistance is mainly due to gene mutations. Specifically, the *cfr* gene belongs to the methylated transferases, which can act on the binding site of linezolid and methylate at position 2503 of the 23S rRNA gene, thus making bacteria resistant to linezolid. The *cfr* gene has been identified in clinical linezolid-resistant Gram-positive bacteria, indicating that the presence of the *cfr* gene is an important mechanism of bacterial resistance to linezolid [8,9].

In multicenter, randomized, double-blind, and non-inferiority trials, the efficacy and safety of tedizolid and linezolid were compared for the treatment of Gram-positive ABSSSI infections (intravenous tedizolid 200 mg once daily for 6 days, or intravenous linezolid 600 mg twice daily for 10 days) [10,11]. In these studies (Establish-1 and Establish-2), clinical response was high (>70%) and similar with both antibiotics.

Although tedizolid and linezolid have a similar safety profile, tedizolid seems to present a lower incidence of gastrointestinal adverse effects and bone marrow suppression than linezolid [12]. Tolerability in clinically important subpopulations (obese, elderly, renal impairment, hepatic disease/impairment) appears to be comparable to the overall population [13].

In order to preserve both new and older antimicrobials, it is critical to evaluate if the dose administered to the patient is sufficient to provide the necessary exposure for optimal clinical response. The success of the therapy depends on the characteristics of the patient, the pharmacokinetic profile of the antibiotic, and the susceptibility of the microorganism responsible for the infection [14]. In fact, inadequate dose selection is one of the most common reasons why drug development programs fail to achieve New Drug Application (NDA) approval [15].

To maximize the likelihood of a favorable clinical/microbiological response, and to minimize the probability of exposure-related toxicity, pharmacokinetic/pharmacodynamic (PK/PD) modelling represents a very useful tool for dose regimen decision-making. The use of Monte Carlo simulation provides an estimate of the antibiotic dosing regimen’s probability of achieving the targeted pharmacodynamic exposure, given uncertainty in patient pharmacokinetics and the minimum inhibitory concentration (MIC) distribution of the bacterial population [16]. The study of PK/PD is an iterative process whereby pre-clinical (in vitro and in vivo) experiments, population PK models, and in silico simulations are used to investigate potential dosing regimens and PK/PD targets. These studies can be used to inform regimen selection for clinical studies [17]. In the field of antibacterial agents, the guidelines of the European Medicine Agency (EMA, Amsterdam, The Netherlands) and its Committee for Medicinal Products for Human Use (CHMP, London, UK) on the evaluation of medicinal products include PK/PD studies [18].

PK/PD analysis integrates information about the concentration of the drug that reaches the infection site and induces the therapeutic response, and the susceptibility of the pathogen against the antibiotic, expressed as the MIC. The quantitative relationship between a pharmacokinetic parameter and a microbiological parameter is known as a PK/PD index. For tedizolid and linezolid, the PK/PD index that best correlates with efficacy is the ratio of the area under the free drug concentration–time curve at steady state over 24 h to the MIC (*f*AUC_24_/MIC). The magnitude required for antimicrobial efficacy has been established as 3 for tedizolid and 80 for linezolid [19,20,21].

In this work, we evaluated by PK/PD analysis and Monte Carlo simulation the activity of tedizolid and compared it to linezolid, with the objective of predicting the probability of treatment success considering the susceptibility profile of staphylococci, enterococci, and streptococci reported in Europe and the United States.

## 2. Results

**Figure 1:**
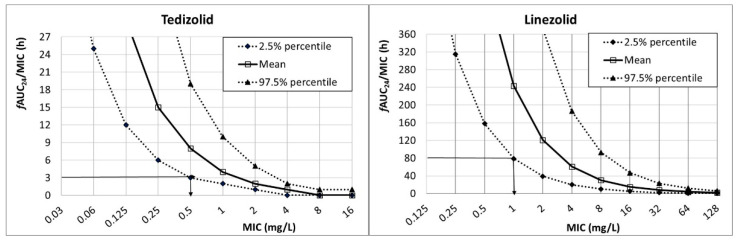
Relationship between *f*AUC_24_/MIC and MIC for tedizolid (200 mg q24h) and linezolid (600 mg q12h).

Figure 1 shows the *f*AUC_24_/MIC for tedizolid (200 mg q24h) and linezolid (600 mg q12). The PK/PD breakpoint, which is the highest MIC value at which there is a high probability of target attainment, can be read directly from the figure at the intersection of the horizontal line at the PK/PD target and the lower limit of the 95% confidence interval (2.5% percentile). For tedizolid (PK/PD target: *f*AUC_24_/MIC ≥ 3), the PK/PD breakpoint was 0.5 mg/L, and for linezolid (PK/PD target: *f*AUC_24_/MIC ≥ 80) it was 1 mg/L.

Table 1 features the PTA values of both antimicrobials for an MIC range from 0.12 to 16 mg/L. PTA is defined as the probability that the specific value of the PK/PD index associated with the efficacy is achieved at a certain MIC value. In other words, it corresponds to the percentage of simulated patients with the estimated PK/PD index equal to or higher than the value related to efficacy against a pathogen with a certain MIC [14]. Simulation results confirm that for the selected targets, standard doses of tedizolid and linezolid cover infections with MIC values up to 0.5 and 1 mg/L, respectively.

Table 2, Table 3 and Table 4 show the susceptibility pattern of tedizolid and linezolid for staphylococci, enterococci, and streptococci compiled from different studies who reported MIC data of these microorganisms for the two antibiotics. The tables include resistance phenotype, number of isolates, collection period, and antimicrobial activity. The rates of isolates inhibited at every MIC value were used for Monte Carlo simulations.

Monte Carlo simulation allowed us to estimate the cumulative fraction of response (CFR), defined as the expected population PTA for a specific drug dose and a specific population of microorganisms. This index must be understood as the expected probability of success of a dosing regimen against bacteria in the absence of the specific value of MIC, and thus the population distribution of MICs is used. Table 5 shows the probability of success (expressed as CFR) of tedizolid and linezolid for staphylococci considering the susceptibility rates reported in the different studies. CFR values close to 100% were obtained with tedizolid for *S. aureus*, and for all strains of MRSA except linezolid-resistant (LR) isolates reported by Barber et al. [26] and methicillin- and linezolid-resistant *S. aureus* (MLRSA) isolates reported by Peñuelas et al. [27]. CFR with tedizolid was close to 100% for LR CoNS isolates reported by Rodríguez-Avial et al. [24]. Linezolid only provided CFR values higher than 90% for CoNS strains from the study of Zurenko et al. [23].

Table 6 features the CFR values of tedizolid and linezolid for enterococci. For *E. faecalis* and other enterococci, tedizolid provided CFR ranging from 98% to 100%. Regarding *E. faecium*, CFR was higher than 85% except for the daptomycin-non-susceptible (DNS) and vancomycin-resistant (VR) isolates from the study of Barber et al. [26] and VR isolates from the study of Pfaller et al. [25]. CFR values obtained for linezolid was 80% when using the MIC distribution provided by Zurenko et al. [23], who reported the susceptibility rates of enterococci without differentiating species, although *E. faecium* and *E. faecalis* were the most represented. When considering the MIC distribution reported by Klupp et al. [28], CFR was 0 for both linezolid and tedizolid. For other enterococci, the CFR obtained for linezolid ranged from 79% to 85%.

The CFR values of both antimicrobials for streptococci are presented in Table 7. Irrespective of the microorganism, the CFR of tedizolid was always 100%, and the CFR of linezolid varied from 91% to 98%.

## 3. Discussion

Linezolid, the first representative member of the oxazolidinone family introduced into the pharmaceutical market, shows excellent activity; however, in recent years, resistance to microorganisms (mainly MRSA) has emerged [31]. Due to the limited number of therapeutic options available for ABSSSIs, new therapeutic alternatives have been developed, especially for resistant Gram-positive microorganisms. In fact, the development of new generations of antimicrobials is one of the most accepted strategies to mitigate the current and future impact of antimicrobial resistance. One of the new antibiotics developed to treat ABSSSIs is tedizolid, which has greater potency, a better spectrum of activity, and a lower resistance profile [2].

PK/PD analysis and Monte Carlo simulation have proved to be very useful tools to select adequate antibiotic treatments with the goal of increasing efficacy and reducing the risk of selecting multidrug-resistant isolates [32]. The use of PK/PD analyses can ameliorate this risk and improve the likelihood of selecting an effective dose regimen, thereby increasing the likelihood of success. These tools have also been applied to identify changes in the antimicrobial activity of antibiotics, providing complementary information to the simple assessment of MIC values [33,34,35], and to establish PK/PD breakpoints [14] as well. 

In this work, we calculated the PK/PD breakpoint of tedizolid and linezolid based on the likelihood of obtaining a targeted exposure. PK/PD breakpoints can be estimated as the highest MIC value at which a high probability of target attainment is obtained (PTA ≥ 90%). Another option to estimate the breakpoints is by graphical representation of the PK/PD index as a function of the MIC [36], which provides a much more restrictive PK/PD breakpoint. In our study, the PK/PD breakpoint for tedizolid was 0.5 mg/L, and it was 1 mg/L for linezolid. For *S. aureus*, the tedizolid PK/PD breakpoint matched the epidemiologic cutoff value ECOFF—that is, the highest MIC for organisms devoid of phenotypically detectable acquired resistance mechanisms [21]. At present, there is no clinical nor ECOFF breakpoint defined for enterococci, so the PK/PD breakpoint may be especially useful when considering this antibiotic as a treatment option. An important advantage of PK/PD breakpoints, as opposed to the clinical ones, is that they are species-independent. In addition, they consider the antibiotic exposure—that is, they are calculated for a specific dosage regimen, and therefore they help to optimize the selection of doses. Regarding streptococci, tedizolid clinical breakpoints have only been defined for β-hemolytic groups A, B, C, and G streptococci and the *S. anginosus* group. For these species, the PK/PD breakpoint agrees with the current clinical breakpoint defined at 0.5 mg/L. Regarding linezolid, the PK/PD breakpoint we calculated (1 mg/L) is lower than the clinical breakpoints (4 mg/L for staphylococci and enterococci, and 2 mg/L for streptococci). However, according to the linezolid-EUCAST rationale document for clinical breakpoints [20], Monte Carlo simulations and target attainment rates for 600 mg twice a day support a susceptible breakpoint of less than 1 or 2 mg/L, which is in agreement with our PK/PD breakpoint. According to Mouton [37], the use of pharmacokinetic parameters from different populations in Monte Carlo simulations for established dosing regimens results in different breakpoints. When discrepancies in breakpoints are observed, the PK/PD breakpoints are generally lower than those defined by the CLSI or EUCAST [32], as seen in the present study. Consequently, and compared to tedizolid, the qualification of an isolate as susceptible for linezolid according to the MIC value and the clinical breakpoint may be less useful in guiding therapy.

The highest MIC values that provided a PTA value ≥90% (0.5 mg/L for tedizolid and 1 mg/L for linezolid, see Table 1) agree with the PK/PD breakpoints considering the EUCAST approach [36]. According to our results, a high probability of treatment success is expected with tedizolid if infection is due to microorganisms with MIC ≤ 0.5 mg/L. Looking through the MIC values reported in Europe and the USA, irrespective of the microorganism (Table 2, Table 3 and Table 4), most isolates would be covered by the standard dose of tedizolid. In the case of linezolid, a high probability of treatment success would be achieved for MIC ≤ 1 mg/L.

CFR is an index that estimates the probability of target attainment for an MIC distribution, and it is very useful to predict the probability of treatment success when applied empirically. For staphylococci (Table 5), tedizolid was adequate for most isolates, including LR, heterogenous vancomycin intermediate (hVISA), vancomycin intermediate (VISA), CoNS, and MRSA. Delpech et al. [38] demonstrated that tedizolid remains active against staphylococci strains harboring the *cfr* gene, probably due the sterically compact nature of the hydroxymethyl group of this drug. The potential spread of *cfr*-mediated linezolid resistance in *S. aureus* makes tedizolid very useful for treating infections due to multidrug-resistant Gram-positive pathogens. Only for MLRSA reported by Peñuelas et al. [27] (18 isolates) and LR CoNS isolates reported by Rodríguez-Avial et al. [24] (164 isolates), the probability of treatment success is low (62% and 20%, respectively). The genetic basis for linezolid resistance in isolates reported by Rodríguez-Avial et al. [24] was not studied, but the susceptibility rates and CFR value indicate that resistance to tedizolid was not related to the *cfr* gene.

Regarding enterococci (Table 6), tedizolid was adequate for *E. faecalis*, regardless of the MIC provided by the different studies (CFR or probability of treatment success of 99–100%). The same is applicable to other enterococci. For *E. faecium*, tedizolid also provides a high probability of treatment success, except for the LR and DNS isolates reported by Barber et al. [26] and the VRE isolates reported by Klupp et al. [28], in which the *cfr* gene was not present. In fact, resistance associated with the *cfr* gene is not predominant in enterococci; alterations in 23S rRNA remain the main oxazolidinone-resistance mechanism in *E. faecium*, while the *optrA* gene prevails in *E. faecalis* [39,40]. Tedizolid represents a therapeutic option only for a limited subset of LR-VRE strains [28]. Regarding linezolid, only Sahm et al. [22] and Zurenko et al. [23] provide susceptibility rates for enterococci, and according to them, linezolid provides a moderate probability of treatment success when used for infections due to enterococci (even VRE and VSE). 

Finally, both tedizolid and linezolid are expected to provide a high probability of treatment success when used for infections due to streptococci (Table 7). These results were expected due to the high susceptibility profile of streptococci to oxazolidinones.

Our results are based on simulations, and a number of considerations are assumed. A previous work [41] reports the main limitations of PK/PD analysis and Monte Carlo simulation. First, pharmacokinetic parameters in the specific population must be used since it is known that pathophysiological conditions affect the distribution and elimination of antimicrobials. In our study, the CL of tedizolid was obtained from pooled data from seven densely and sparsely sampled clinical trials, most subjects were healthy, and the CL of linezolid used was from healthy subjects. Second, pharmacokinetic equations used to estimate drug exposure are usually simple models that facilitate calculations. Third, the simulations in our work were based on serum pharmacokinetics, and therefore the results are mainly applicable to bloodstream infections. Moreover, PK/PD modelling is based on prior studies that have identified correlations between PK/PD indices and health outcomes. These relationships have typically been derived from animal models; however, a good correlation and validation between animal models and the clinic has been established.

## 4. Materials and Methods

This study was performed in different steps: (1) acquisition of pharmacokinetic parameters, PK/PD targets, and susceptibility data; and (2) PK/PD analysis and Monte Carlo simulation. 

### 4.1. Pharmacokinetic Parameters, PK/PD Targets, and Susceptibility Data

The PK parameters of linezolid and tedizolid, as well as the PK/PD targets, were obtained from published studies [19,20,21,42,43]. These data are presented in Table 8.

The CL value of tedizolid was obtained with a population PK model developed with data from four phase 1 studies, one phase 2 study, and three phase 3 studies, in which tedizolid was administered by either intravenous or oral route. The CL of linezolid is for healthy volunteers.

The antimicrobial activity of linezolid and tedizolid for staphylococci, enterococci, and streptococci were obtained from the literature [22,23,24,25,26,27,28,29,30]. We selected those studies that provided MIC values for both antibiotics. Clinical isolates from Europe and the United States were collected between 2011 and 2016. Table 2, Table 3 and Table 4 show the MIC values of staphylococci, enterococci, and streptococci, respectively, obtained from the abovementioned studies. The MIC distribution of the isolates from the different studies was used for the PK/PD analysis.

### 4.2. Pharmacokinetic/Pharmacodynamic Analysis and Monte Carlo Simulation

Monte Carlo simulation is an advanced statistical modeling tool that makes it possible to expand the sample size considering the variability of the PK and PD parameters on the estimation of the PK/PD indices in order to provide predictions of the likely result of different therapeutic approaches, or the achievement of therapeutic targets [14]. A 10,000-subject Monte Carlo simulation was conducted for each antibiotic using Oracle^®^ Crystal Ball Fusion Edition v.11.1.2.3.500 (Oracle USA Inc., Redwood City, CA). The values of *f*AUC_24_/MIC (the ratio of the area under the free drug concentration–time curve at steady state over 24 h to MIC) were calculated for tedizolid (200 mg q24h) and linezolid (600 mg q12h) over an MIC range of serial two-fold dilutions from 0.06 to 16 mg/L and from 0.06 to 128 mg/L, respectively. The following equation was used [32]:

*f*AUC_24_/MIC = D × Fu/CL (1)

where D is the daily dose, Fu is the unbound fraction, and CL is the total clearance.

For simulations, a log-normal distribution was assumed for CL, according to statistical criteria. Unbound fraction was included as a fixed value [44].

For every MIC value and considering the variability of the CL, the output of the simulation consisted of a probability distribution, and the mean value and the 95% CI (expressed as percentiles) of the *f*AUC_24_/MIC were extracted. The PK/PD breakpoint, considered as the highest MIC value at which *f*AUC_24_/MIC is ≥3 or ≥80 for tedizolid and linezolid, respectively, were estimated. According to EUCAST, the PK/PD breakpoint was obtained from the lower limit of the 95% CI (2.5% percentile) [36].

The PTA [45], defined as the probability that a specific value of a PK/PD index associated with the efficacy of the antimicrobial treatment is achieved at a certain MIC, was estimated. PTA ≥80% but <90% was associated with moderate probabilities of success, whereas PTA ≥ 90% was considered as optimal against that bacterial population [32].

*CFR* values, which allowed us to calculate the probability of success of an empiric treatment, were calculated considering the PTA for each MIC value and the bacterial population MIC distribution, according to the following equation [45]:

(2) $$CFR\;\left(\%\right)=\overset{n}{\underset{i=1}{\sum }}PTA_{i}\cdot F_{i}$$

where *i* indicates the MIC category, *PTA_i_* is the PTA of each MIC category, and *F_i_* is the fraction of the microorganism population in each MIC category. As with PTA, CFR ≥80% but <90% was associated with moderate probabilities of success, whereas CFR ≥ 90% was considered as optimal against that bacterial population [32].

## 5. Conclusions

In conclusion, PK/PD breakpoints calculated for tedizolid and linezolid were 0.5 and 1 mg/L, respectively. For empiric treatment, tedizolid seems to be adequate for the treatment of infections due to most staphylococci, enterococci, and streptococci. For LR isolates not related to the *cfr* gene, tedizolid does not guarantee efficacy. This study confirms the importance of considering the susceptibility profile of the geographical area or hospital setting and the PK/PD analysis to guide empiric therapy.

## Figures and Tables

**Table 1:** Probability of target attainment (PTA) for tedizolid (200 mg q24h) and linezolid (600 mg q12h).

		Probability of Target Attainment, PTA (%)								
		MIC (mg/L)								
	PK/PD Target	0.06	0.125	0.25	0.5	1	2	4	8	16
Tedizolid	*f*AUC_24_/MIC ≥ 3	100	100	100	97	55	11	1	0	0
Linezolid	*f*AUC_24_/MIC ≥ 80	100	100	100	100	97	62	19	4	0

In grey PTA values ≥ 90% (high probability of success).

**Table 2:** Resistance phenotype, number of isolates, collection period, and antimicrobial activity of staphylococci for tedizolid and linezolid.

Microorganism/Study	Antimicrobial	Resistance Phenotype	Nº Isolates	Collection Period	Percent of Isolates (%) Inhibited at MIC (mg/L) of:							
					≤0.12	0.25	0.5	1	2	4	8	>8
***S. aureus***												
Sahm et al. [22]	Tedizolid		4499	2011–2012	10.8	55.2	33.9					
	Linezolid					0.2	0.3	27.2	67.8	4.2	0.0	0.3
Zurenko et al. [23]	Tedizolid		7187	2008–2013	7.7	62.3	29.8	0.2	0.1	0.1		
	Linezolid					0.1	0.3	21.6	72.8	4.9	0.0	0.2
Rodríguez-Avial et al. [24]	Tedizolid	LR	5	2010–2011			100					
	Linezolid										60.0	40.0
Pfaller [25]	Tedizolid		4364	2014–2015	95.2	4.8						
**MRSA**												
Sahm et al. [22]	Tedizolid		1770	2011–2012	12.7	53.2	33.8	0.1	0.1	0.1		
	Linezolid					0.2	0.3	30.8	65.7	2.7	0.0	0.3
Barber et al. [26]	Tedizolid	hVISA	120	Before 2016	20.8	45.9	31.6	1.7				
		VISA	100		59.0	25.0	16.0					
		DNS	75		30.7	50.6	18.7					
		LR	7		28.6	28.5	0	42.9				
Peñuelas et al. [27]	Tedizolid	MRSA	18	Before 2016	11.1	61.1	27.8					
		MLRSA				11.1	11.1	72.2	5.6			
	Linezolid	MRSA	18					38.9	61.1			
		MLRSA									16.7	83.3
Pfaller et al. [25]	Tedizolid		1006	2014–2015	96.6	3.4						
**CoNS**												
Sahm et al. [22]	Tedizolid		537	2011–2012		51.4	39.1	8.6	0	0	0.7	
Zurenko et al. [23]	Tedizolid		674	2008–2013	47.9	40.6	10.0	0.7	0.0	0.6	0.2	
	Linezolid					0.9	23.2	61.2	12.6	1.2	0.1	0.8
Rodríguez-Avial et al. [24]	Tedizolid	LR	164	2010–2011	1.2	2.5	12.2	6.1	7.3	49.4	20.1	1.2
	Linezolid	LR									4.3	95.7
Pfaller et al. [25]	Tedizolid		729	2014–2015	98.6	1.2	0	0	0.1			

Light grey: values under the susceptibility range. DNS: daptomycin-non-susceptible; LR: linezolid resistant; hVISA: heterogeneous vancomycin intermediate; VISA: vancomycin intermediate; MRSA: methicillin-resistant *S. aureus*, MLRSA: methicillin- and linezolid-resistant *S. aureus*.

**Table 3:** Resistance phenotype, number of isolates, collection period, and antimicrobial activity of enterococci for tedizolid and linezolid.

Microorganism/Study	Antimicrobial	Resistance Phenotype	Nº Isolates	Collection Period	Percent of Isolates (%) Inhibited at MIC (mg/L) of:							
					≤0.12	0.25	0.5	1	2	4	8	>8
***E. faecalis***												
Sahm et al. [22]	Tedizolid		634	2011–2012	5.8	54.8	39.1	0.1	0.2			
Barber et al. [26]	Tedizolid		100	Before 2016	30.0	69.0	1.0					
Pfaller et al. [25]	Tedizolid		559	2014–2015	55.6	44.0	0.4					
***E. faecium***												
Sahm et al. [22]	Tedizolid		221	2011–2012	10.4	52.5	35.7	0.9	0.5			
Barber et al. [26]	Tedizolid		120	Before 2016	5.0	42.5	26.7	20.8	2.5	2.5		
		LR	10					40.0	30.0	30.0		
		DNS	25			44.0	12.0	32.0	8.0	4.0		
Pfaller et al. [25]	Tedizolid		311	2014–2015	80.4	18.6	0.6	0.0	0.3			
		VRE	245		79.2	20.0	0.8					
		VSE	66		84.8	13.6	0.0	1.5				
					**MIC (mg/L)**							
Klupp et al. [28]					**2**	**4**	**8**	**16**	**32**	**64**	**128**	**256**
	Tedizolid	VRE	30	2012–2015	20.0	60.0	10.0	3.3	6.7			
	Linezolid	VRE							26.7	20.0	20.0	33.3
***Enterococcus* spp. (predominantly *E. faecalis* and *E. faecium* without differentiating species)**					**MIC (mg/L)**							
					**≤0.12**	**0.25**	**0.5**	**1**	**2**	**4**	**8**	**>8**
Zurenko et al. [23]	Tedizolid		1241	2008–2013	5.7	50.7	42.2	1.0	0.3	0.1		
	Linezolid					0.4	2.7	40.7	54.4	1.4	0.1	0.3
**Other enterococci**												
Sahm et al. [22]	Tedizolid		18	2011–2012	33.3	38.9	27.8					
	Tedizolid	VRE	163		10.4	52.2	36.2	0.6	0.6			
	Linezolid	VRE					1.2	40.5	57.2	0.5	0	0.6
	Tedizolid	VSE	705		14.2	73.2	12.6					
	Linezolid	VSE			0.1	0.5	5.9	51.9	41.3	0.3		

Light grey: values under the susceptibility range. DNS: daptomycin-non-susceptible; LR: linezolid resistant; VRE: vancomycin resistant; VSE: vancomycin susceptible.

**Table 4:** Resistance phenotype, number of isolates, collection period, and antimicrobial activity of streptococci for tedizolid and linezolid.

Microorganism/Study	Antimicrobial	Resistance Phenotype	Nº Isolates	Collection Period	Percent of Isolates (%) Inhibited at MIC (mg/L) of:						
***S. pneumoniae***					**0.094**	**0.125**	**0.19**	**0.25**	**0.38**		
Hipp et al. [29]	Tedizolid	Penicillin-susceptible	56	2009–2016	1.8	3.6	57.1	35.7	1.8		
					**MIC (mg/L)**						
					**0.064**	**0.125**	**0.25**	**0.5**	**1**	**2**	**4**
EUCAST [30]	Linezolid		60,180			0.1	1.0	7.8	65.3	25.9	
					**MIC (mg/L)**						
Pfaller et al. [25]					**0.015**	**0.03**	**0.06**	**0.12**	**0.25**	**0.5**	**1**
	Tedizolid		1273	2014–2015	0.3	0.5	6.4	72.5	20.2		
***Streptococcus* spp.**					**MIC (mg/L)**						
Zurenko et al. ^a^ [23]					**0.06**	**0.12**	**0.25**	**0.5**	**1**	**2**	**4**
	Tedizolid		1600	2008–2013	5.00	44.38	50.13	0.50			
	Linezolid						3.50	26.13	65.13	5.19	0.06
***S. anginosus* group**											
Zurenko et al. ^b^ [23]	Tedizolid		91	2008–2013	38.46	43.96	17.58				
	Linezolid						38.46	35.16	26.37		
***S.* viridans group**											
Pfaller et al. ^c^ [25]	Tedizolid		218	2014–2015	38.53	59.17	2.29				
**β-hemolytic streptococci**										
Pfaller et al. ^d^ [25]	Tedizolid			2014–2015	93.5	4.5					

^a^ Includes *S. pyogenes*, *S. agalactiae*, S. groups C, F, and G, and *S.* viridans group; ^b^ Includes isolates of *S. anginosus*, *S. intermedius*, and *S. constellatus*; ^c^ Includes *S. anginosus*, *S. anginosus* group, *S. australis*, *S. constellatus*, *S. cristatus*, *S. gordonii*, *S. infantis*, *S. lutetiensis*, *S. massiliensis*, *S. mitis*, *S. mitis* group, *S. mitis/oralis*, *S. mutans*, *S. oralis*, *S. parasanguinis*, *S. salivarius*, *S. salivarius* group, and *S. sanguinis*. ^d^ Includes *S. agalactiae*, *S. canis*, *S. dysgalactiae*, and *S. pyogenes*. Light grey: values under the susceptibility range.

**Table 5:** Probability of success (expressed as CFR or cumulative fraction of response) of tedizolid and linezolid for staphylococci.

Microorganism/Study	Resistance Phenotype	Probability of Success (CFR, %)	
		Tedizolid	Linezolid
***S. aureus***			
Sahm et al. [22]		99	69
Zurenko et al. [23]		99	67
Rodríguez-Avial et al. [24]	LR	97	3
Pfaller et al. [25]		100	n.c.
**MRSA**			
Sahm et al. [22]			71
Barber et al. [26]	hVISA	99	n.c.
	VISA	100	n.c.
	DNS	100	n.c.
	LR	80	n.c.
Peñuelas et al. [27]	MRSA	99	75
	MLRSA	62	n.c.
Pfaller et al. [25]		100	n.c.
**CoNS**			
Sahm et al. [22]		94	n.c.
Zurenko [23]		99	93
Rodríguez-Avial et al. [24]	LR	20	0
Pfaller et al. [25]		100	n.c.

DNS: daptomycin-non-susceptible; LR: linezolid resistant; hVISA: heterogenous vancomycin intermediate; VISA: vancomycin intermediate; MRSA: methicillin-resistant *S. aureus,* MLRSA: methicillin- and linezolid-resistant *S. aureus*. n.c.: not calculated due to lack of MIC data.

**Table 6:** Probability of success (expressed as CFR) of tedizolid and linezolid for enterococci.

Microorganism/Study	Resistance Phenotype	Probability of Success (CFR, %)	
		Tedizolid	Linezolid
***E. faecalis***			
Sahm et al. [22]		99	n.c.
Barber et al. [26]		100	n.c.
Pfaller et al. [25]		100	n.c.
***E. faecium***			
Sahm et al. [22]		99	n.c.
Barber et al. [26]		86	n.c.
	LR	25	n.c.
	DNS	75	n.c.
Klupp et al. [28]	VRE	0	0
Pfaller et al. [25]		100	n.c.
	VRE	100	n.c.
	VSE	99	n.c.
***Enterococcus* spp.** (predominantly *E. faecalis* and *E. faecium*)			
Zurenko et al. [23]		98	80
**Other enterococci**			
Sahm et al. [22]		99	n.c.
	VRE	98	79
	VSE	100	85

DNS: daptomycin-non-susceptible; LR: linezolid resistant; VRE: vancomycin resistant; VSE: vancomycin susceptible. n.c.: not calculated due to lack of MIC data.

**Table 7:** Probability of success (expressed as CFR or cumulative fraction of response) of tedizolid and linezolid for streptococci.

Microorganism/Study	Resistance Phenotype	Probability of Success (CFR, %)	
		Tedizolid	Linezolid
***S. pneumoniae***			
			
Hipp et al. [29]	Penicillin-susceptible	100	n.c.
EUCAST et al. [30]			91
Pfaller [25]		100	n.c.
***Streptococcus* spp.**			
Zurenko et al. [23]		100	98
***S. anginosus* group**			
Zurenko et al. [23]		100	98
***S.* viridans group**			
Pfaller et al. [25]		100	n.c.
**β-hemolytic streptococci**		
Pfaller et al. [25]		100	n.c.

n.c.: not calculated due to lack of MIC data.

**Table 8:** Pharmacokinetic parameters and PK/PD targets of tedizolid and linezolid used for Monte Carlo simulations. CL data are expressed as mean ± standard deviation.

Antibiotic	Dosing Regimen	CL (L/h)	Fu	PK/PD Target	References
Tedizolid	200 mg q24h	6.69 ± 2.07	0.10	*f*AUC_24_/MIC ≥ 3	[19,21,42]
Linezolid	600 mg q12h	4.80 ± 1.74	0.69	*f*AUC_24_/MIC ≥ 80	[19,20,43]

CL: total body clearance; Fu: unbound fraction; fAUC24: area under the unbound concentration–time curve over a period of 24 h.

## Data Availability

All data are applicable in the paper.
